# Efficacy of probiotic supplements in the treatment of sarcopenia: A systematic review and meta-analysis

**DOI:** 10.1371/journal.pone.0317699

**Published:** 2025-02-06

**Authors:** Yi Wang, Ping Lei

**Affiliations:** College of Integrative Medicine, Liaoning University of Traditional Chinese Medicine, Shenyang, China; Hong Kong Baptist University, HONG KONG

## Abstract

Although some studies have confirmed the efficacy of probiotics in the treatment of sarcopenia, the intervention of sarcopenia is a comprehensive consideration of many factors, and the efficacy of probiotics is still controversial. Therefore, this study systematically evaluated the efficacy of probiotics in the intervention of sarcopenia via high—quality meta—analysis, providing a basis for the clinical diagnosis and treatment of sarcopenia. Randomized controlled trials related to probiotics in the treatment of sarcopenia were searched in PubMed, Embase, the Cochrane Library and Web of Science. The search time was from inception to 2024-07-17. Two investigators independently screened the articles, extracted data, and assessed the risk of bias of the included studies. Meta-analysis was performed using RevMan 5.4 and Stata 14.0 software. A total of 22 eligible studies were included. The results showed that there was no statistically significant difference between probiotics and placebo in improving muscle mass and Lean body mass in sarcopenia patients; MD: 0.66, 95%CI: - 0.01–1.33; Z = 1.93, *P* > 0.05; MD: - 0.13, 95%CI: -0.81–0.55; Z = 0.38, *P* = 0.71 > 0.05. However, probiotics were found to significantly improve overall muscle strength compared with the placebo group. MD: 2.99, 95%CI: 2.14–3.85; Z = 6.86, *P* < 0.001. Probiotics can significantly improve global muscle strength in patients with sarcopenia. It is suggested that probiotics have certain clinical value in the clinical treatment of sarcopenia, but the results may be limited by the number and quality of included studies. The above conclusions need to be verified by more high-quality studies.

## Introduction

Sarcopenia is a geriatic syndrome characterized by age-related loss of muscle mass, decreased muscle strength and/or decreased body function [1], It was first proposed by American Professor Rosenberg in 1989. The prevalence of sarcopenia in Asia ranges from 5.5% to 25.7% [2]. By 2050, the global elderly population will reach 2 billion, and the number of patients with sarcopenia will continue to increase in the future, gradually becoming a new public problem in the world, causing serious global health burden [3]. Sarcopenia is a growing health problem among older patients. Early diagnosis is important to intervene and improve muscle status as well as body function [4]. Recent studies have shown that sarcopenia may be improved by the gut-muscle axis, suggesting that gut health may influence muscle phenotypes [5]. Based on the gut-muscle axis, several studies have reported positive changes in gut microbiota composition induced by probiotics, suggesting a link between gut health and muscle homeostasis [6,7].

Probiotics can have a direct or indirect impact on various aspects of health by altering the composition of the gut microbiome and producing bioactive metabolites [8]. *Lactic acid* bacteria are currently the most studied probiotic strains, which have anti-inflammatory, anticancer and antioxidant effects. In addition, it contributes to immune enhancement, reduction of oxidative stress, and improvement of intestinal barrier function [9,10]. Study has found that HY7602 *Lactobacillus curvatus* by increasing muscle mass, muscle fibers regeneration and mitochondrial biology, improve muscle to reduce disease and athletic performance [11]. Study suggested that *L*. *rhamnosus* IDCC3201 represented a promising dietary supplement with the potential to alleviate sarcopenia by modulating the gut microbiome and metabolites[8]. The above studies have confirmed the effectiveness of probiotics on sarcopenia.

In recent years, a large number of clinical studies have reported the therapeutic advantages of probiotics in this disease, including many randomized controlled trials (RCTS). RCTS are internationally recognized as the gold standard for evaluating interventions. Although some studies have confirmed the efficacy of probiotics in the treatment of sarcopenia, the intervention of sarcopenia is a comprehensive consideration of many factors, and the efficacy of probiotics is still controversial. Therefore, this study systematically evaluated the efficacy of probiotics in the intervention of sarcopenia via high—quality meta—analysis, providing a basis for the clinical diagnosis and treatment of sarcopenia.

## Data and methods

### Data and sources

The study has been registered on the Prospero International System Evaluation Registration Platform and the registration number was CRD42024571445. The first author was responsible for searching PubMed, Embase, the Cochrane Library, Web of Science databases. The search terms mainly include “muscle atrophy” OR “Atrophies, Muscular” OR “sarcopenia” OR “Muscular Atrophies” And “Probiotics” OR “Probiotic preparations” OR “Probiotic supplements” OR “Synbiotics” OR “Bifidobacterium” OR “Bacillus” OR “Lactobacillus” OR “Saccharomycetes” OR “Clostridium” OR “Escherichia” And “randomized controlled trial” OR “randomized controlled trial” OR “Random” OR “clinical trial” OR “Trial” OR “Efficacy” OR “Clinical” etc.”. The search time was from inception of each database to 2024-July 17.

### Inclusion and exclusion criteria

Inclusion criteria: ① Patients with definite diagnosis of sarcopenia; ②Age ≥ 18 years old, regardless of gender or race; ③ The control group was treated with placebo, and the experimental group was treated with probiotics; (4) Study type: randomized controlled study; ⑤ At least one outcome index was included, including Muscle Mass, Lean body mass, grip strength, gait speed, etc.

Exclusion criteria: ① Reviews, case reports, conference articles, individual cases or non-clinical studies; ② Studies where data could not be extracted; ③ The subjects had no definite diagnosis of sarcopenia; ④ Duplicate publications; ⑤ Non-English articles.

### Research data extraction

Two researchers independently screened the articles, extracted the data and cross-checked them. If there is any disagreement, it shall be resolved through consultation with the third party personnel. The data extraction content mainly includes: (1) the basic information of the included research (name of the first author, publication time, research region, etc.); (2) Basic information of the research subject (sample size, age, etc.); (3) Outcome indicators and related data; (4) Key elements of bias risk assessment.

### Research quality assessment

The risk of bias in the included studies was independently evaluated by two investigators, the results were cross-checked, and differences were resolved in consultation with third parties. According to the Risk Bias assessment tool 5.4 of the Cochrane Evaluator Manual, two researchers were used to evaluate the generation of random sequences in the included studies, the blind method of allocation, the blind method used in the study process, the blind method used in the study outcome measurement, the integrity of the result data, the existence of selective publication and other biases. Select "high risk of bias" when the implementation method is wrong, select "low risk of bias" when the implementation method is correct, select "unknown bias" when there is no description of this item.

The risk of bias was assessed on the following seven dimension: Randomization sequence generation (selection bias); allocation concealment (selection bias); Blinding of participants and personnel (performance bias); Blinded of outcomes assessment (detection bias); Incomplete outcome data (attrition bias); Selective reporting (reporting bias); and other biases. Each dimension was classified as having a high, low, or uncertain risk. If all seven items of the included studies were at low risk of bias, the quality of the studies were rated as grade A. If there was no high risk of bias but there was an uncertain risk of bias, it was rated grade B. If an item had a high risk of bias, it was rated grade C.

### Statistical analysis

Statistical analysis was performed using RevMan5.4 and Stata 14.0 software. Relative risk (RR) was used as the effect size for binary variables, and mean difference (MD) was used as the effect size for continuous variables. standardized mean difference (SMD) was used for different measurement units. Each effect size is given its point estimate and 95% confidence interval (CI). The χ2 test was used to test the heterogeneity of the experimental results, and when there was no significant statistical heterogeneity (*P* > 0.1, I^2^ ≤ 50%), the fixed effect model was used for analysis. When statistical heterogeneity was suggested (*P* < 0.1, I^2^ > 50%), sensitivity analysis was used to further analyze the source of heterogeneity. When no significant heterogeneity was found, the random effects model was used for analysis. Publication bias was assessed using funnel plot and Egger’s test. *P* < 0.05 was considered as significant difference.

## Result analysis

### Search results

**Fig 1:**
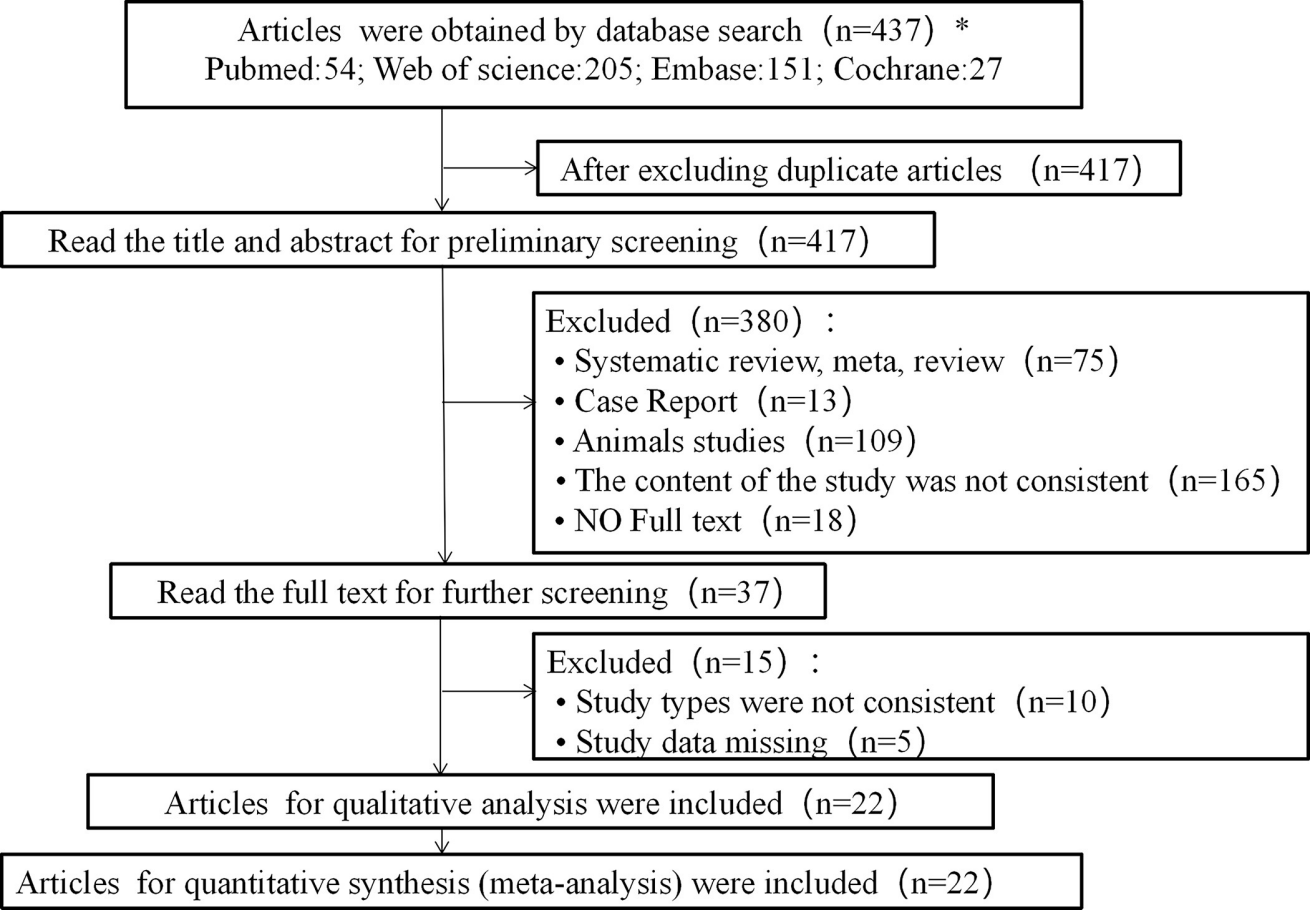
Flowchart of articles screening.

By searching the relevant articles in major databases, re-screening the subject, abstract and full text of the articles, 22 RCTs were obtained. The flow chart of article selection was shown in (Fig 1).

### Study characteristics

A total of 22 studies involving 1028 patients were included, with intervention periods ranging from 3 to 48 weeks. Four studies were conducted in Taiwan of China [5,12–14]; three in Japan [15–17], three in South Korea [18–20], two in Poland [21,22], two in the USA [23,24], one in India [25], one in Thailand [26], one in China [27], one in Iran [28], one in Pakistan [29], one in Sweden [30], one in Slovakia [31], and one in Estonia [32]. For details, see (Tables 1 and 2).

**Table 1:** Study and participant characteristics of the included studies.

Study (Year)	Country and region	Study Type	Health Status Or complications	Probiotics VS Placebo (n Male/Female)	Probiotics (Age)	Placebo (Age)
Chaiyasut 2022 [26]	Thailand	RCT	Healthy	24 (3/21) VS 24 (7/17)	61.6 (0.8)	58.8 (1.2)
Karim 2022 [29]	Paksitan	RCT	Patients with chronic heart failure	44 (44/0) VS 48 (48/0)	67.6 (4.9)	65.2 (5.6)
Tarik 2022 [25]	India	RCT	Healthy (resistance trained)	28 (28/0) VS 28 (28/0)	21.1 (2.5)	20.9 (3.1)
Sohn 2021 [20]	South korea	RCT	Overweight	35 VS 36	47.8 (11.7)	45.5 (10.0)
Lee 2021 b [14]	Taiwan of China	RCT	Healthy	8 (8/0) VS 8 (8/0)	24.6 (2.8)	25.6 (4.1)
Lee 2021 c [33]	Taiwan of China	RCT	Frail	13 (8/5) VS 17 (9/8)	80.5 (9.4)	75.2 (7.2)
Hric 2021 [31]	Slovakia	RCT	Healthy	13 (0/13) VS 9 (0/9)	51 (12.6)	44 (13.0)
Hajipoor 2021 [28]	Iran	RCT	Obese	28 VS 31	40.9 (6.8)	35.4 (11.7)
Lim 2020 [19]	South korea	RCT	Overweight and obese	47 VS 48	46.4 (12.2)	47.2 (11.2)
Pan 2020 [27]	China	RCT	Metabolic syndrome and type 2 diabetes	15 (7/8) VS 16 (8/8)	53.6 (6.8)	57.6 (6.1)
Huang 2019 a [12]	Taiwan of China	RCT	Healthy	18 (9/9) VS 18 (9/9)	Males 22.0 (1.7) Females 23.0 (5.2)	Males 22.4 (1.8) Females 20.8 (1.0)
Huang 2019 b [13]	Taiwan of China	RCT	Healthy (Triathletes)	9 VS 9	20.2 (0.7)	21.1 (1.5)
Skrypnik 2019 [21]	Poland	RCT	Obese	23 (0/23) VS 24 (0/24)	56.0 (6.6)	60.5 (6.9)
Inoue 2018 [15]	Japan	RCT	Healthy	20 (7/13) VS 18 (7/11)	69.9(3.0)	70.9 (3.2)
Nilsson 2018 [30]	Sweden	RCT	Low Bone mineral density	32 (0/32) VS 36 (0/36)	76.4 (1.0)	76.3 (1.1)
Minami 2018 [16]	Japan	RCT	Overweight	40 (37/3) VS 40 (37/3)	45.4 (9.8)	45.6 (8.5)
Szulinska 2018 [22]	Poland	RCT	Obese	23 (0/23) VS 24 (0/24)	55.2 (6.9)	58.7 (7.3)
Toohey 2020 [23]	USA	RCT	Healthy (Volleyball, soccer athletes)	11 (0/11) VS 12 (0/12)	19.6 (1.0)	19.6 (1.0)
Kim 2018 [18]	South Korea	RCT	Overweight and obese	26 VS 25	37.9 (34.7–41.2)	38.1 (34.1–42.2)
Osterberg 2015 [24]	USA	RCT	Healthy	9 (9/0) VS 11 (11/0)	22.4 (1.4)	22.9 (0.9)
Minami 2015 [17]	Japan	RCT	Overweight	19 (6/13) VS 25 (11/14)	58.9 (2.0)	61.9 (1.9)
Sharafedtinov 2013 [32]	Estonia	RCT	Metabolic syndrome and hypertension	25 (9/16) VS 11	52.0 (10.9)	51.7 (12.1)

**Table 2:** Treatment features included in the study.

Study (Year)	Dose	Duration (week)	Intervention	Control	Outcomes indicators
Chaiyasut 2022 [26]	2 x 10^10^ (*Lactobacillus paracasei* Hll01. *Bifidobacterium breve*) and 10^10^ (*Bifidobacterium longum)* CFU	12 weeks	*L*. *paracasei* Hl101. *B*.*breve* *B*. *longum*	Placebo	Muscle mass
Karim 2022 [29]	11.2 x 10^10^ CFU	12 weeks	*Bifidobacteria* (*B*.*longum* DSM24736, *B*.*breve* DSM24732, 24737) and *Lactobacillus* (DSM 24735, DSM 24730, DSM 24733 *Lactobacillus delbrueckii* subsp. *Bulgaricus* DSM24734) and *Streptococcus thermophilus* (DSM 24731)	Placebo	Muscle mass Global muscle strength
Tarik 2022 [25]	2 x10^9^ CFU	8 weeks	*Bacillus coagulans* and 20 g whey protein	Placebo and 20 g whey protein and 4x/week RT	Lean Body Mass Global muscle strength
Sohn 2021 [20]	4 x 10^9^ CFU	12 weeks	*Lactobacillus plantarum* K50 and advice on exercise and healthy eating	Placebo and advice on exercise 3x/week and healthy eating	Lean Body Mass
Lee 2021 b [14]	20 g	4 weeks	Synkefir containing *L*. *paracasei* DSM32785 (LPC12). *Lactobacillus rhamnosus* DSM 32786 (LRH10), *Lactobacillus helveticus* DSM 32787 (LH43), *Lactobacillus fermentum* DSM 32784 (LF26) and *S*. *the rmophilus* DSM 32788 and exercise	Placebo and exercise at 60–80% VO_2_ max	Muscle mass
Lee 2021 c [33]	6 x 10^10^ CFU	18 weeks	*L*. *plantarum* TWK10	Placebo	Lean Body Mass Global muscle strength
Hric 2021 [31]	30 g	4 weeks	Bryndza cheese and weight loss and concurrent training	Regular cheese and weight loss and concurrent training	Muscle mass
Hajipoor 2021 [28]	4x10^7^ CFU each strain	10 weeks	Probiotic low-fat yogurt 100g (*Lactobacillus acidophilus* La-B5 *Bifidobacterium lactis* Bb-12) and low-calorie diet	Low-fat yogurt 100 g and low-calorie diet	Lean Body Mass
Lim 2020 [19]	10 x 10^10^ CFU	12 weeks	*Lactobacillus sakei* (CJLS03) and exercise and healthy eating advice	Placebo and exercise and healthy eating advice	Lean Body Mass
Pan 2020 [27]	Males 90 g Females 75 g	8 weeks	Fermented noodles (*L*. *plantarum*)	Wheat noodles	Muscle mass
Huang 2019 a [12]	9 x 10^10^ CFU	6 weeks	*L*. *Plantarum* TW10K	Placebo	Muscle mass
Huang 2019 b [13]	6 x 10^10^ CFU	4 weeks	*L*. *plantarum* PS128 and concurrent training	Placebo and training	Muscle mass
Skrypnik 2019 [21]	10^10^CFU	12 weeks	*Bifidobacterium bifidum* W23, *B*.*lactis* W51. *B*. *lactis* W52. *Lactobacillus acidophilus* W37. *Lactobacillus brevis* W63. *Lactobacillus casei* W56, *Lactobacillus salivarius* W24. *Lactococcus lactis* W19 and *Llactis* W58	Placebo	Lean Body Mass
Inoue 2018 [15]	1.25 x 10^10^ CFU each strain	12 weeks	*longum* BB536. *Bifidobacterium infantis* M-63, *B*.*breve M-16V* and *B*. *breve B-3* and RT	Placebo	Lean Body Mass
Nilsson 2018 [30]	10^10^ CFU	48 weeks	*Lactobacillus reuteri*	Placebo	Lean Body Mass
Minami 2018 [16]	2 x10^9^ CFU	12 weeks	*B*. *breve* B-3	Placebo	Muscle mass
Szulinska 2018 [22]	10^10^ CFU	12 weeks	*B*.*bifidum* W23. *B*.*lactis* w51. *B*.*lactis* w52, *L*.*acidophilus* W37. *L*.*brevis* W63. *L*.*casei* W56. *L*.*salivarius* W24, *L*.*lactis* W19 and *L*.*lactis* w58	Placebo	Lean Body Mass
Toohey 2020 [23]	5 x 10^9^CFU	10 weeks	*Bacillus subtilis* (DE111) and recovery drink (45 g CHO, 20 g protein, 2 g fat)	Placebo and recovery drink	Lean Body Mass Global muscle strength
Kim 2018 [18]	10^10^ CFU	12 weeks	*Lactobacillus gasseri* BNR17 and mild energy restriction and increased physical activity	Placebo and mild energy restriction and physical activity	Lean Body Mass
Osterberg 2015 [24]	9 x 10^10^ CFU	4 weeks	*S*.*thermophilus* DSM24731. *L*.*acidophilus* DSM24735. *L*.*delbrueckii ssp*. Bulgaricus DSM24734, *L*.*paracasei* DSM24733, *L*.*plantarum* DSM24730. *B*.*lonqum* DSM24736.8. infantis DSM24737 and *B*.*breve* DSM24732 and high-fat and hypocaloric diet	Placebo	Lean Body Mass
Minami 2015 [17]	5 x 10^10^ CFU	12 weeks	*B*.*breve* B-3	Placebo	Muscle mass
Sharafedtinov 2013 [32]	50 g to 1.5 x 10^11^ CFU	3 weeks	Cheese (*L*.*plantarum* TENSlA) and hypocaloric diet	Placebo and hypocaloric diet	Muscle mass

### Study quality evaluation

**Fig 2:**
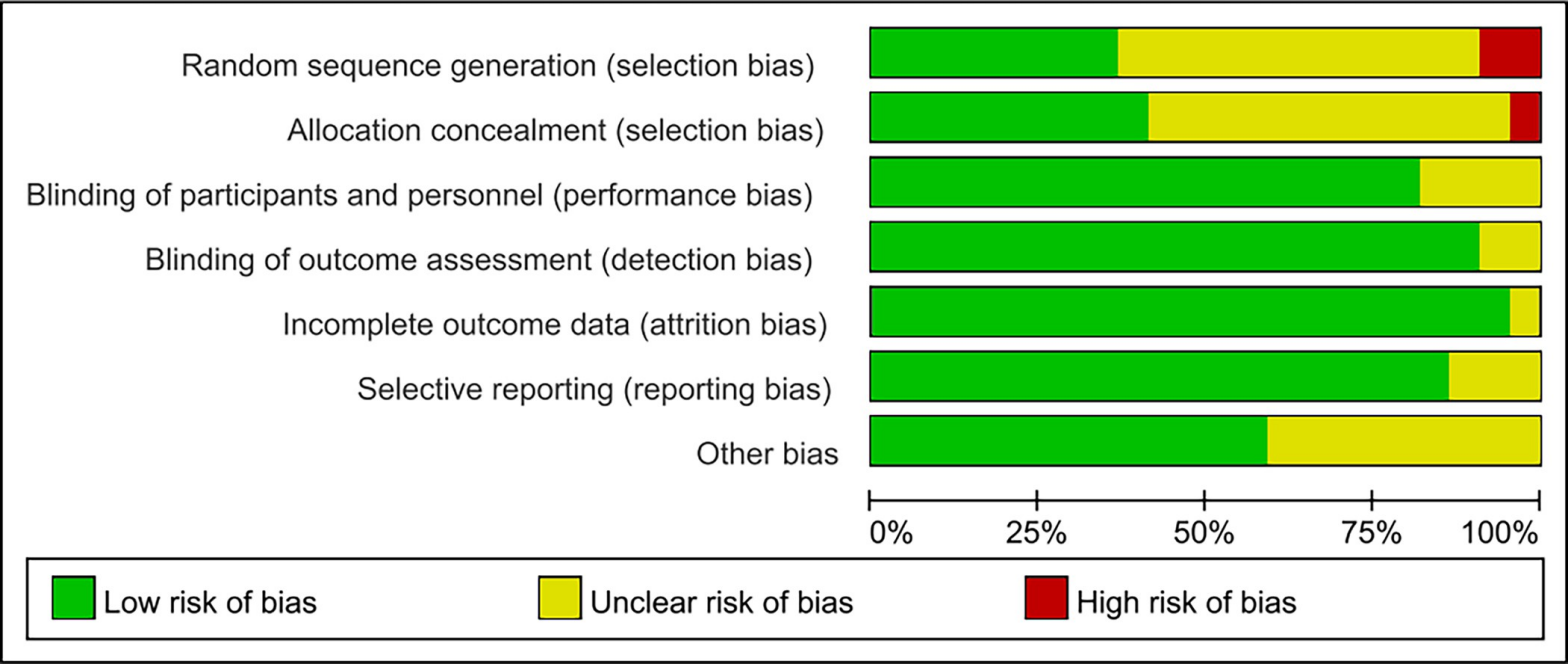
Risk of bias graph.

**Fig 3:**
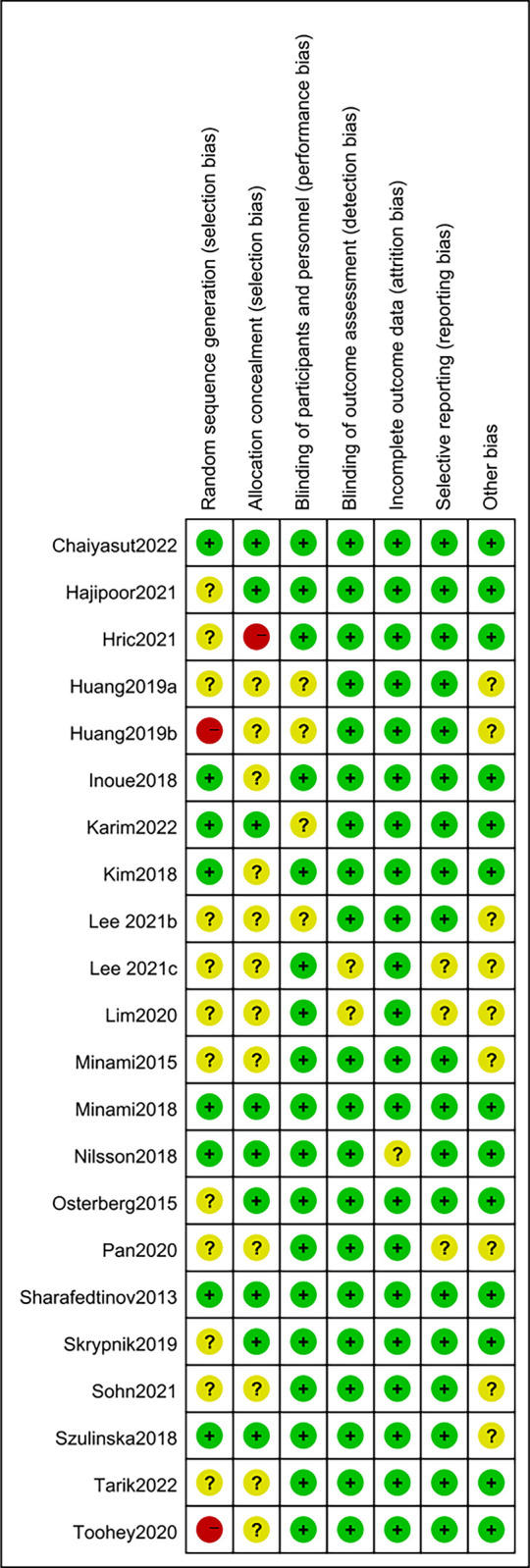
Risk of bias summary.

Revman 5.4 software was used to draw the risk of bias assessment chart. Overall, the quality of the studies was moderate. The results of study quality evaluation showed that three studies were grade A [16,26,32], sixteen studies were grade B [5,12,14,15,17–22,24,25,27–30], and three studies were grade C [13,23,31]. A study by Toohey et al. [23] did not mentioned random allocation and did not describe the method and implementation strategy of random allocation in detail, so it was judged as " high ". The study of Huang et al. [13] did not mention the generation of random sequences, so it was judged as " high ". The studies of Huang et al. [12] and Lee et al. [5] did not mention randomization, allocation hiding and blind method, so they were judged to be "unclear". See (Figs 2 and 3).

## Results of meta-analysis

### Muscle mass

**Fig 4:**
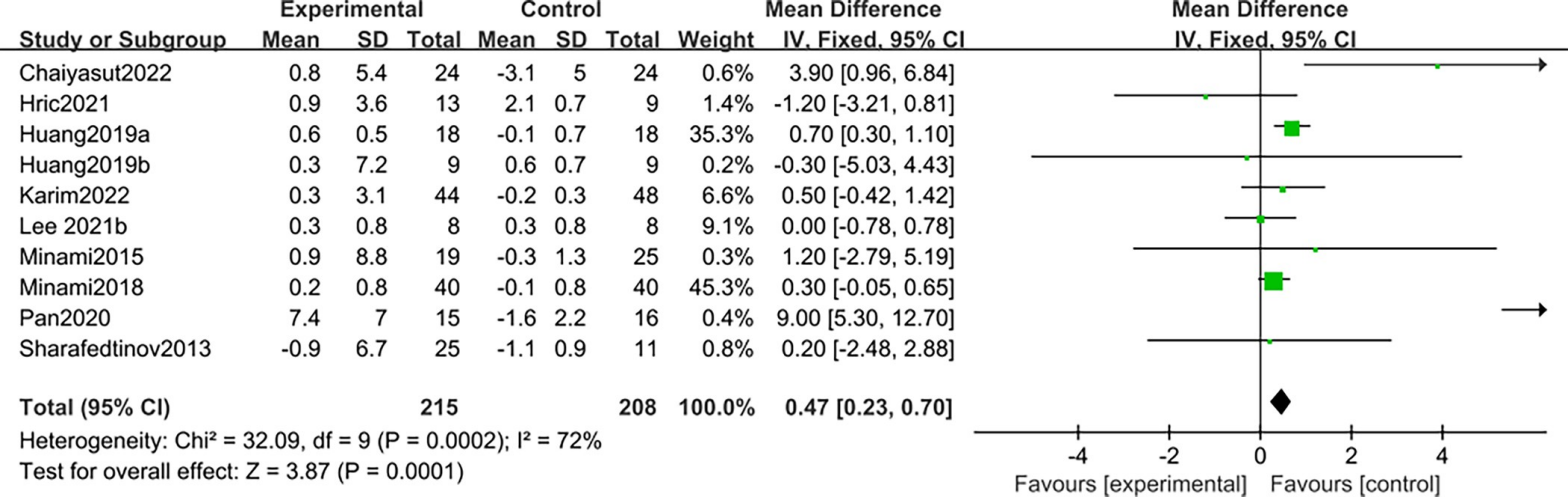
Effect of probiotics on muscle mass in patients with sarcopenia.

Ten studies [12–14,16,17,26,27,29,31,32] reported the effect of probiotics on muscle mass in patients with sarcopenia. Due to the large heterogeneity (I^2^ = 72%, *P*<0.1), a random effects model was selected. It was found that there was no statistically significant difference between probiotics and placebo in improving muscle mass in patients with sarcopenia; MD: 0.66, 95%CI: -0.01–1.33; Z = 1.93, *P* > 0.05. See (Fig 4).

**Fig 5:**
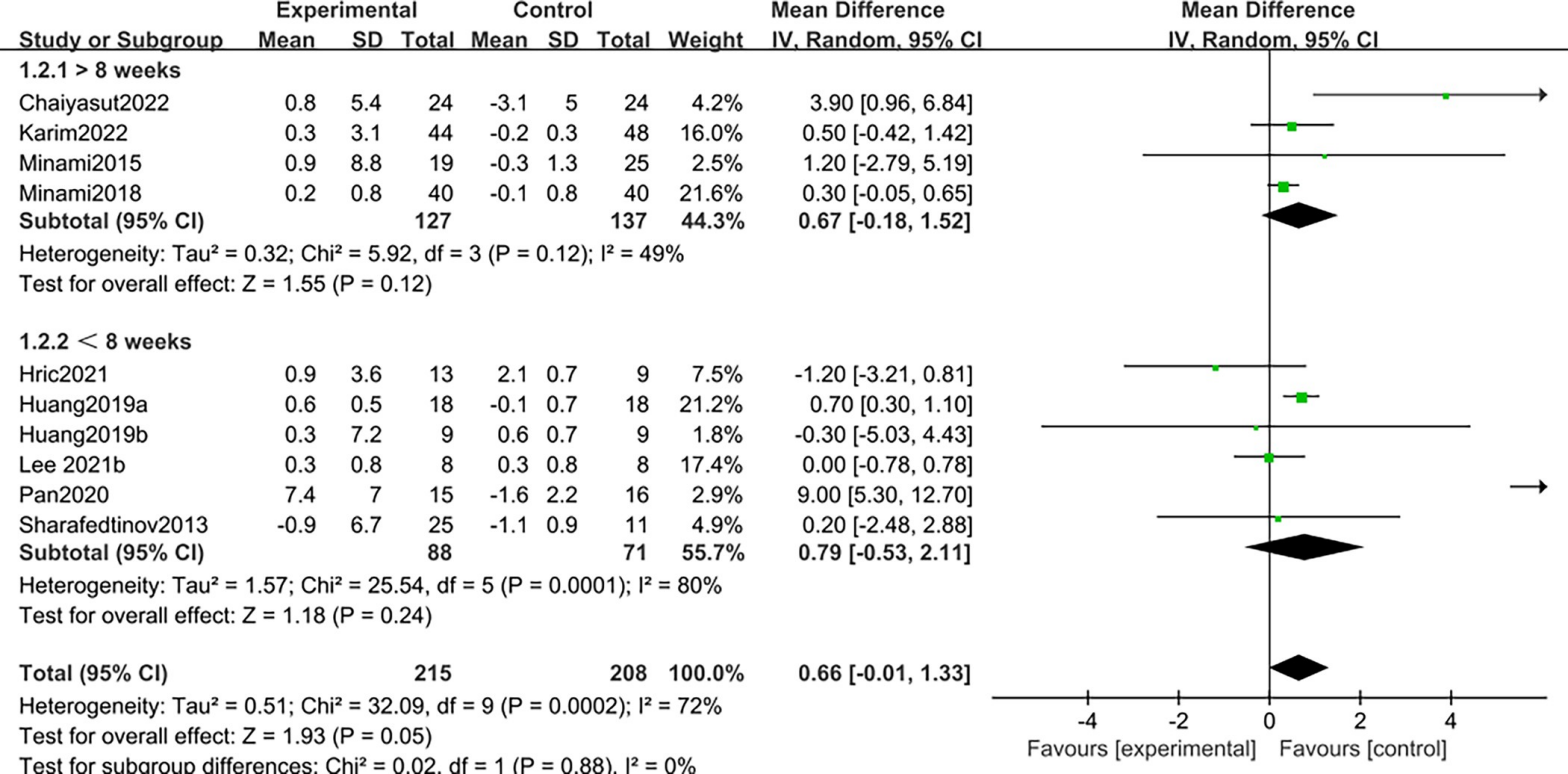
Subgroup analysis was carried out according to the period of study.

Due to the large heterogeneity, subgroup analysis was performed based on whether the study period was longer than 8 weeks, and the results showed that the study intervention cycle > 8 weeks had a larger impact on overall studies. Respectively, <8 weeks [12–14,27,31,32], Z = 1.18, *P* = 0.24> 0.05, I^2^ = 80%; >8 weeks [16,17,26,29]; Z = 1.55, *P* = 0.12 > 0.05, I^2^ = 49%; Overall Z = 1.93, *P* = 0.05 > 0.05. See (Fig 5) for details.

**Fig 6:**
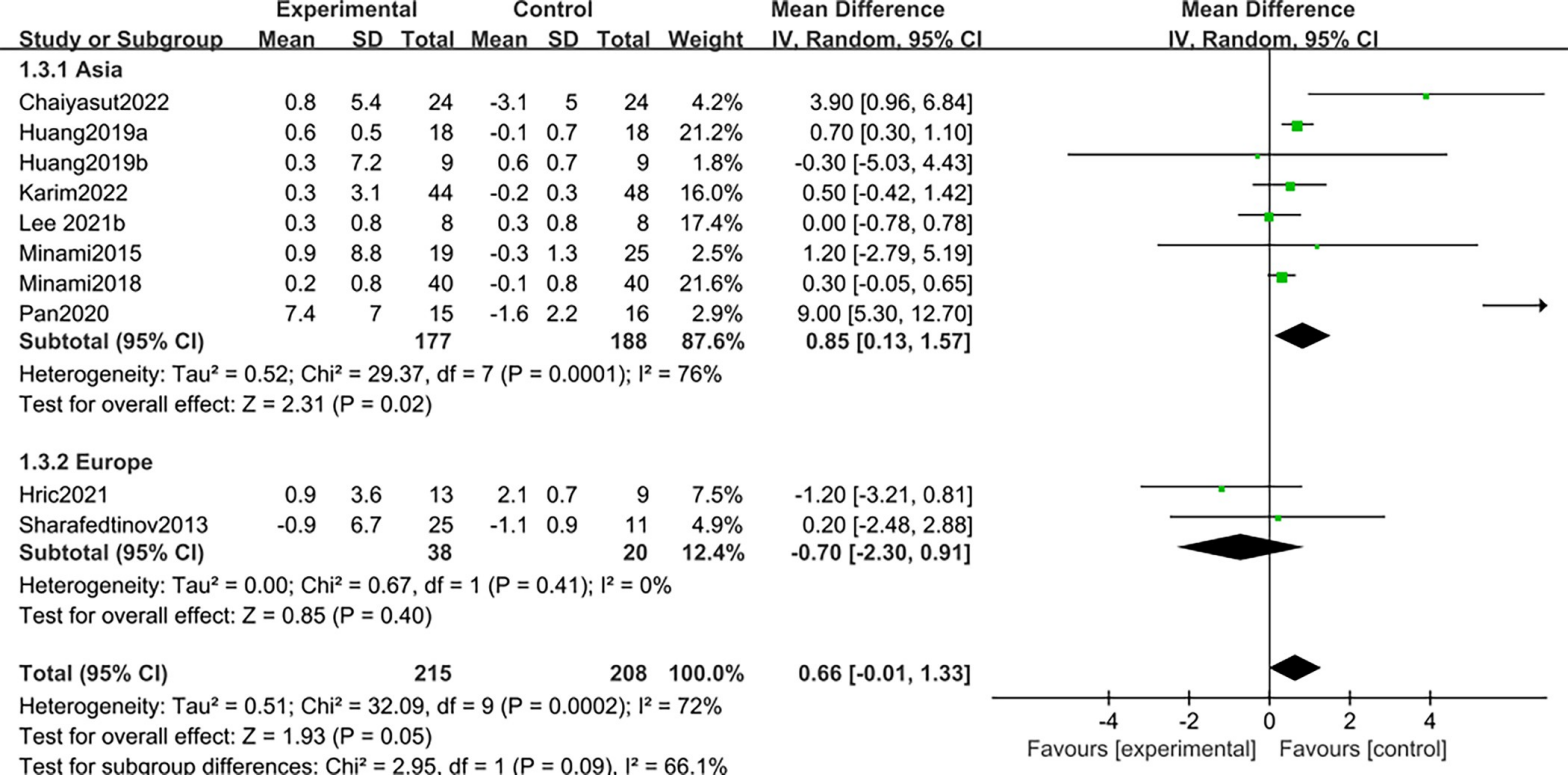
Subgroup analysis based on the country in which the study was conducted.

At the same time, a subgroup analysis was performed based on the countries in which the study was conducted, and the results found that studies from Asia had a greater impact on the overall study. Respectively, Europe [31,32], Z = 0.85, *P* = 0.40 > 0.05, I^2^ = 0%; Asia [12–14,16,17,26,27,29], Z = 2.31, *P* = 0.02, I^2^ = 76%; Overall Z = 1.93, *P* = 0.05 > 0.05; See (Fig 6) for details.

### Lean body mass

**Fig 7:**
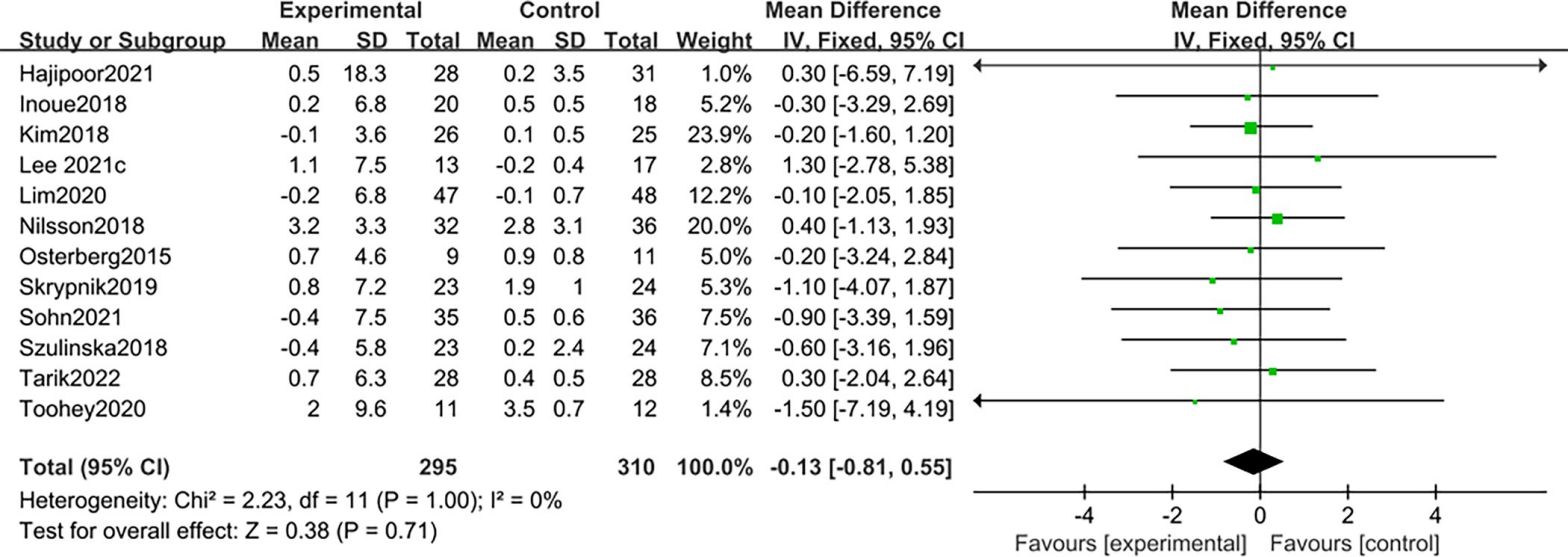
Effect of probiotics on lean body mass in patients with sarcopenia.

There were 12 articles [15,18–25,28,30,33] reported the effect of probiotics on lean body mass, and the fixed effects models were used due to the small heterogeneity (I^2^ = 0%, *P* > 0.1). The results found that probiotics did not significantly improve lean body mass compared with the placebo group. MD: - 0.13, 95%CI: -0.81–0.55; Z = 0.38, *P* = 0.71 > 0.05. See in (Fig 7).

### Global muscle strength

**Fig 8:**
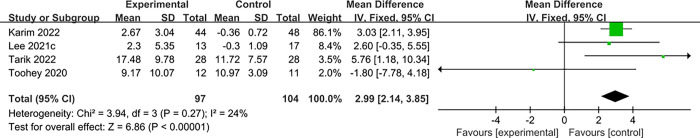
Effect of probiotics on global muscle strength in patients with sarcopenia.

There were 4 articles [14,23,25,29] reported the effect of probiotics on global muscle strength, and fixed effects models were used due to the small heterogeneity (I^2^ = 24%, *P* > 0.1). The results found that probiotics significantly improved global muscle strength compared with the placebo group. MD: 2.99, 95%CI: 2.14–3.85; Z = 6.86, *P* < 0.001. See in (Fig 8).

### Handgrip strength

Two studies [27,33] assessed handgrip strength, but due to different data types, further pooling could not be carried out.

## Analysis of publication bias

**Fig 9:**
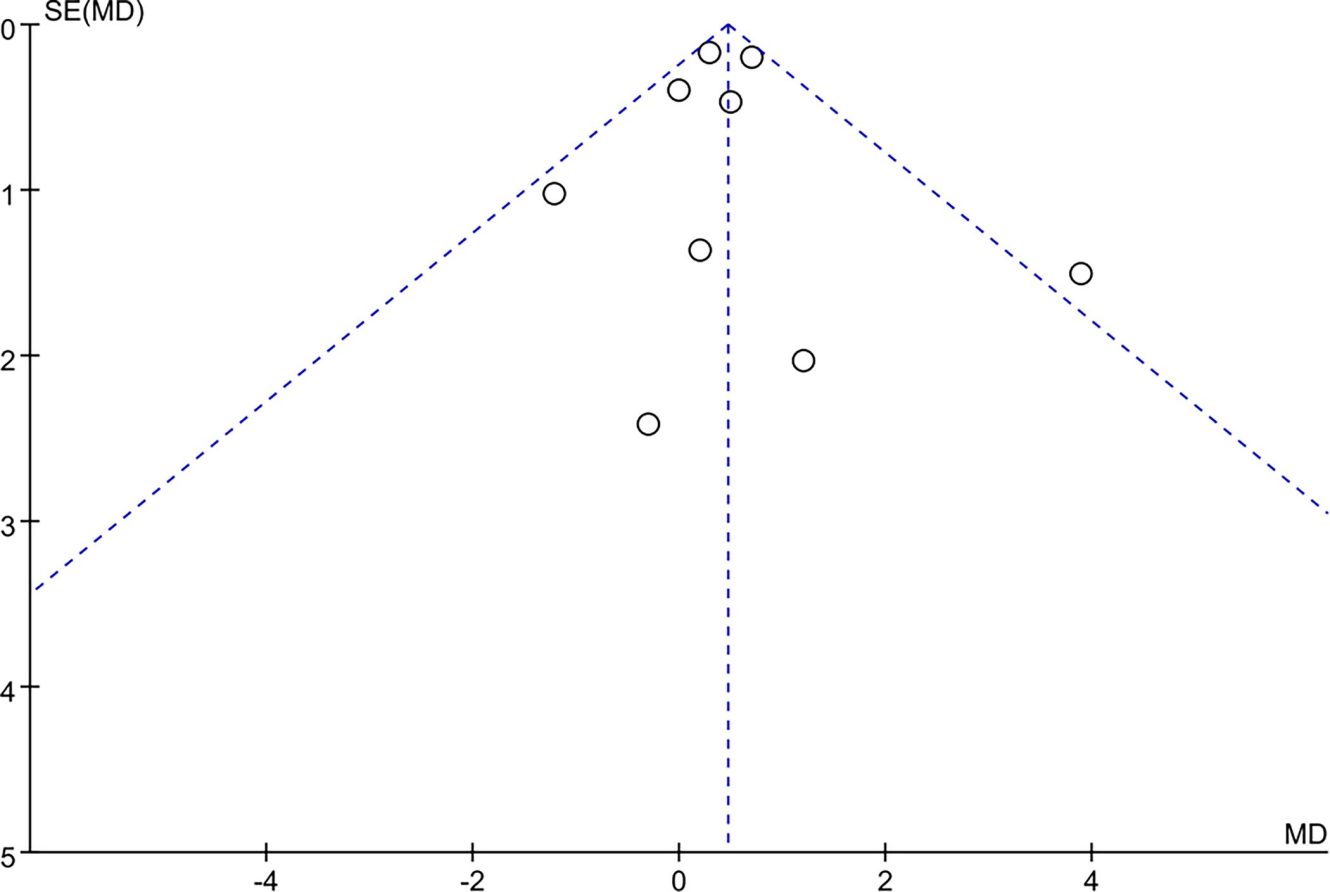
Publication bias graph of muscle mass.

**Fig 10:**
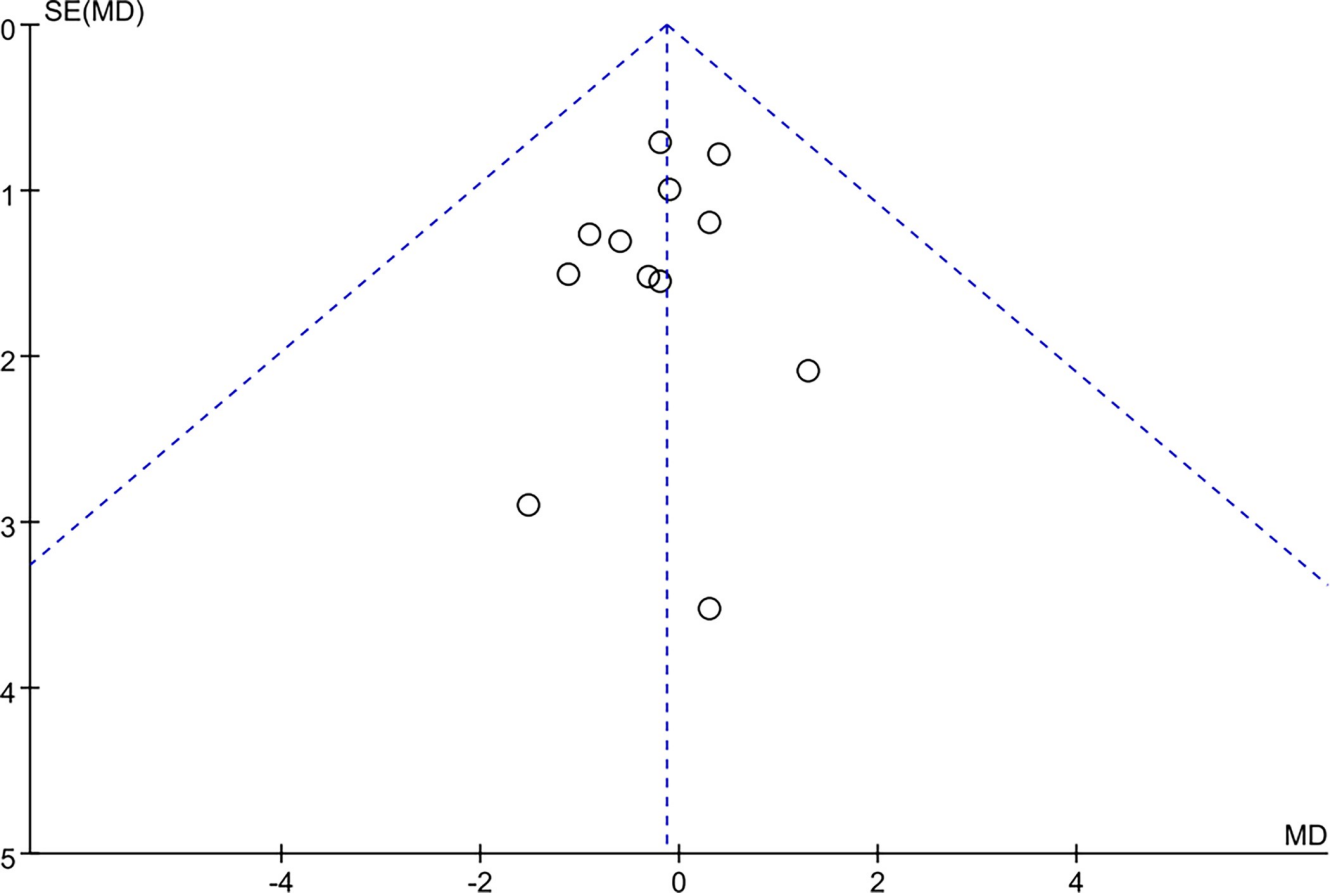
Publication bias graph of Lean body mass.

Funnel plot and Egger’s test were used to evaluate publication bias of outcome indicators of muscle mass and Lean body mass. The results showed that the distribution of all studies in the main outcome indicators was roughly symmetrical, Respectively, *P* = 0.333 > 0.05, *P* = 0.838 > 0.05; suggesting no significant publication bias. See (Figs 9 and 10).

## Discussion

Sarcopenia is a multifactorial syndrome associated with skeletal muscle aging, which undergoes morphological and physiological changes associated with loss of strength, mass, and function. Sarcopenia increases the risk of frailty, falls, and fractures, leading to disability in the elderly, seriously reducing the quality of life, and increasing hospitalization and mortality rates. Sarcopenia has become a major health problem for the elderly worldwide in the future. It is necessary to identify the high-risk population of sarcopenia early and intervene in time.

The International Scientific Association for Probiotics and Prebiotics defines probiotics as "living microorganisms that, when given in sufficient amounts, are capable of confering health benefits to the host." Human probiotic microorganisms mainly include *bifidobacteriu*m, *Lactobacillus*, *Lactococcus*, yeast, etc. Their advantage is that they can have an impact on the microbiota inhabiting the human body to ensure a proper balance between pathogens and bacteria. The gut-muscle axis has been proposed as a promising target for the alleviation of muscle atrophy. Many factors influence the composition and function of the gut microbiota. In addition, communication between the microbiota and the host is bidirectional, subtle, and continuous. The potential role of the gut microbiome in muscle health is enormous but not fully understood. For example, the microbiota can synthesize tryptophan, an essential amino acid that promotes protein anabolism, and convert tryptophan into a range of metabolites with key metabolic and immune functions [34]. Previous studies have shown that supplementation with probiotic *Lactobacillus plantarum* TWK10 leads to muscle fiber and muscle strength [35] and physical endurance [36]. Similarly, other probiotics have shown beneficial effects; For example, heat-killed *Bifidobacterium* breve B-3 increases muscle mass [37]. While *Lactobacillus plantarum* improves muscle endurance. The above studies have confirmed the effectiveness of probiotics on sarcopenia.

The results found that there was no statistically significant difference between probiotics and placebo in improving muscle mass and Lean body mass in sarcopenia patients; MD: 0.66, 95%CI: - 0.01–1.33; Z = 1.93, *P* > 0.05; MD: - 0.13, 95%CI: - 0.81–0.55; Z = 0.38, *P* = 0.71 > 0.05. However, we found that probiotics significantly improved Global muscle strength compared with the placebo group. MD: 2.99, 95%CI: 2.14–3.85; Z = 6.86, *P* < 0.001. The results of a meta-analysis by Handajani YS et al. [38] found that probiotic treatment improved muscle mass and its endurance compared with placebo. The results of our study were not consistent with theirs. Through further reading of the article, it was found that in terms of muscle mass index, the studies we included were the same as theirs. There were no differences in sample size, study design, probiotic species and dose. And the reason for the inconsistency may be differences in statistical analysis (such as decimal point retention number, etc.). It is suggested that future studies should further refine the data related to different types of probiotics, doses, or the profile of the study population (age, severity of sarcopenia) to explain these differences.

At the same time, a subgroup analysis was conducted based on the countries in which the study was conducted, and the results found that studies from Asia had a larger impact on overall studies and the results showed that the study intervention cycle > 8 weeks had a larger impact on overall studies. The reason for this difference may be related to the dose of oral probiotics, as well as the type of probiotics and the treatment cycle. After further reading the article, it can be found that the studies conducted in Asia have more probiotic species, longer intervention cycles, and larger sample sizes. Moreover, compared with Europe, the differences in ethnicity and physical fitness between the two regions may have contributed to the difference.

Diet is known to affect the gut microbiota and the serum metabolome in adults. For many years, the processed and red meats, fried foods, and sugar-sweetened beverages of the typical Western diet were considered unhealthy. In contrast, the traditional diets of Asians are often associated with a low incidence of chronic diseases. Antioxidants are commonly found in fresh vegetables and fruits. Most countries in Asia have a favorable climate and a large variety of fruits and vegetables, especially green leafy vegetables, which have more varieties than European, American and even Mediterranean countries. Different races, gender, age, physical condition, and even differences in diet, work and rest may affect the effect of probiotics in the human body. Targeted probiotic products should be launched for all ages and different stages of life to adapt to the development of the concept of probiotics in the world.

In conclusion, this study further confirms the effectiveness of probiotics for sarcopenia, but further high-quality studies are needed to support this conclusion. Probiotics play a positive role in regulating the host microbiota, and supplementation of probiotics can effectively alleviate the adverse effects of sarcopenia and improve the health status of patients. Therefore, further study on the application of probiotics in nutritional supplement and health management of patients with sarcopenia is of great significance for maintaining their health.

## Limitation of the study

There are some limitations in this study: (1) The included sample size is small, which may limit the accuracy of the study results. (2) Most of the study comes from the Asian region. It is not known whether ethnicity and different social environment influence treatment effects. (3) The results of meta-analysis showed that there was significant heterogeneity in some study results. The source of heterogeneity was tested by subgroup analysis, but the partial heterogeneity was still high after subgroup analysis. (4) At present, the number of published studies is limited and the quality is not good, and most studies lack descriptions of allocation hiding and implementation blindness, resulting in a high risk of bias in included studies, resulting in a low level of evidence in this study.

## Conclusions

In short, probiotics can significantly improve global muscle strength in patients with sarcopenia. It is suggested that probiotics have certain clinical value in the clinical treatment of sarcopenia, but the results may be limited by the number and quality of included studies. However, more well-designed, large sample, multi-center and multi-investment RCTS are still needed to confirm the results. Future studies are recommended to refine data related to probiotic use, dosage, or study population characteristics (age, severity of sarcopenia).

## Supporting information

S1 File (DOC)

S1 Data (XLSX)

## Abbreviations

RR: Relative risk
MD: mean difference
SMD: standardized mean difference
CI: confidence interval
RCTs: randomized controlled trials
