# Correction: Efficacy of probiotic supplements in the treatment of sarcopenia: A systematic review and meta-analysis

**DOI:** 10.1371/journal.pone.0320199

**Published:** 2025-03-06

**Authors:** Yi Wang, Ping Lei

The Funding statement for this paper is incorrect. The correct Funding statement is as follows: This research is supported by the Natural Science Foundation of Liaoning Province (No. 2019-ZD-0444), the “Yuanzhi” Project of Liaoning University of Traditional Chinese Medicine, and the “Qiji” Project of the College of Integrative Chinese and Western Medicine at Liaoning University of Traditional Chinese Medicine.
