# Supplementary material for: Efficacy of probiotic supplements in the treatment of sarcopenia: A systematic review and meta-analysis
Source: PLoS One. 2025 Feb 6;20(2):e0317699. doi: 10.1371/journal.pone.0317699 (PMC11801621; doi:10.1371/journal.pone.0317699)
Supplement: S1 File — (DOC) [file pone.0317699.s001.doc]

**Table1. Study and participant characteristics of the included studies.**

| **Study (Year)** | **Country and region** | **Study Type** | **Health Status Or complications** | **Probiotics VS Placebo (n Male/Female)** | **Probiotics (Age)** | **Placebo (Age)** |
| --- | --- | --- | --- | --- | --- | --- |
| Chaiyasut 2022 | Thailand | RCT | Healthy | 24 (3/21) VS 24 (7/17) | 61.6 (0.8) | 58.8 (1.2) |
| Karim 2022 | Paksitan | RCT | Patients with chronic heart failure | 44 (44/0) VS 48 (48/0) | 67.6 (4.9) | 65.2 (5.6) |
| Tarik 2022 | India | RCT | Healthy （resistance trained） | 28 (28/0) VS 28 (28/0) | 21.1 (2.5) | 20.9 (3.1) |
| Sohn 2022 | South korea | RCT | Overweight | 35 VS 36 | 47.8 (11.7) | 45.5 (10.0) |
| Lee 2021 b | Taiwan of China | RCT | Healthy | 8 (8/0) VS 8 (8/0) | 24.6 (2.8) | 25.6 (4.1) |
| Lee 2021 c | Taiwan of China | RCT | Frail | 13 (8/5) VS 17 (9/8) | 80.5 (9.4) | 75.2 (7.2) |
| Hric 2021 | Slovakia | RCT | Healthy | 13 (0/13) VS 9 (0/9) | 51 (12.6) | 44 (13.0) |
| Hajipoor 2020 | Iran | RCT | Obese | 28 VS 31 | 40.9 (6.8) | 35.4 (11.7) |
| Lim 2020 | South korea | RCT | Overweight and obese | 47 VS 48 | 46.4 (12.2) | 47.2 (11.2) |
| Pan 2020 | China | RCT | Metabolic syndrome and type 2 diabetes | 15 (7/8) VS 16 (8/8) | 53.6 (6.8) | 57.6 (6.1) |
| Huang 2019 a | Taiwan of China | RCT | Healthy | 18 (9/9) VS 18 (9/9) | Males 22.0 (1.7)  Females 23.0 (5.2) | Males 22.4 (1.8)  Females 20.8 (1.0) |
| Huang 2019 b | Taiwan of China | RCT | Healthy （Triathletes） | 9 VS 9 | 20.2 (0.7) | 21.1 (1.5) |
| Skrypnik 2019 | Poland | RCT | Obese | 23 (0/23) VS 24 (0/24) | 56.0 (6.6) | 60.5 (6.9) |
| Inoue 2018 | Japan | RCT | Healthy | 20 (7/13) VS 18 (7/11) | 69.9(3.0) | 70.9 (3.2) |
| Nilsson 2018 | Sweden | RCT | Low Bone mineral density | 32 (0/32) VS 36 (0/36) | 76.4 (1.0) | 76.3 (1.1) |
| Minami 2018 | Japan | RCT | Overweight | 40 (37/3) VS 40 (37/3) | 45.4 (9.8) | 45.6 (8.5) |
| Szulinska 2018 | Poland | RCT | Obese | 23 (0/23) VS 24 (0/24) | 55.2 (6.9) | 58.7 (7.3) |
| Toohey 2020 | USA | RCT | Healthy (Volleyball, soccer athletes) | 11 (0/11) VS 12 (0/12) | 19.6 (1.0) | 19.6 (1.0) |
| Kim 2018 | South Korea | RCT | Overweight and obese | 26 VS 25 | 37.9 (34.7-41.2) | 38.1 (34.1-42.2) |
| Osterberg 2015 | USA | RCT | Healthy | 9 (9/0) VS 11 (11/0) | 22.4 (1.4) | 22.9 (0.9) |
| Minami 2015 | Japan | RCT | Overweight | 19 (6/13) VS 25 (11/14) | 58.9 (2.0) | 61.9 (1.9) |
| Sharafedtinov 2013 | Estonia | RCT | Metabolic syndrome and hypertension | 25 (9/16) VS 11 | 52.0 (10.9) | 51.7 (12.1) |

**Table 2. Treatment features included in the study.**

| **Study (Year)** | **Dose** | **Duration (week)** | **Intervention** | **Control** | **Outcomes indicators** |
| --- | --- | --- | --- | --- | --- |
| Chaiyasut 2022 | 2 x 10^10^ (*Lactobacillus paracasei* Hll01. *Bifidobacterium breve*) and 10^10^ (*Bifidobacterium longum)* CFU | 12 weeks | *L. paracasei* Hl101.  *B.breve*  *B. longum* | Placebo | Muscle mass |
| Karim 2022 | 11.2 x 10^10^ CFU | 12 weeks | *Bifidobacteria* (*B.longum* DSM24736, *B.breve* DSM24732, 24737) and *Lactobacillus* (DSM 24735, DSM 24730, DSM 24733 *Lactobacillus delbrueckii* subsp. *Bulgaricus* DSM24734) and *Streptococcus thermophilus* (DSM 24731) | Placebo | Muscle mass  Global muscle strength |
| Tarik 2022 | 2 x10^9^ CFU | 8 weeks | *Bacillus coagulans* and 20 g whey protein | Placebo and 20 g whey protein and 4x/week RT | Lean Body Mass  Global muscle strength |
| Sohn 2022 | 4 x 10^9^ CFU | 12 weeks | *Lactobacillus plantarum* K50 and advice on exercise and healthy eating | Placebo and advice on exercise 3x/week and healthy eating | Lean Body Mass |
| Lee 2021 b | 20 g | 4 weeks | Synkefir containing *L. paracasei* DSM32785 (LPC12). *Lactobacillus rhamnosus* DSM 32786 (LRH10), *Lactobacillus helveticus* DSM 32787 (LH43), *Lactobacillus fermentum* DSM 32784 (LF26) and *S. the rmophilus* DSM 32788 and exercise | Placebo and exercise at 60-80% VO_2_ max | Muscle mass |
| Lee 2021 c | 6 x 10^10^ CFU | 18 weeks | *L. plantarum* TWK10 | Placebo | Lean Body Mass  Global muscle strength |
| Hric 2021 | 30 g | 4 weeks | Bryndza cheese and weight loss and concurrent training | Regular cheese and weight loss and concurrent training | Muscle mass |
| Hajipoor 2020 | 4x10^7^ CFU each strain | 10 weeks | Probiotic low-fat yogurt 100g (*Lactobacillus acidophilus* La-B5 *Bifidobacterium lactis* Bb-12) and low-calorie diet | Low-fat yogurt 100 g and low-calorie diet | Lean Body Mass |
| Lim 2020 | 10 x 10^10^ CFU | 12 weeks | *Lactobacillus sakei* (CJLS03) and exercise and healthy eating advice | Placebo and exercise and healthy eating advice | Lean Body Mass |
| Pan 2020 | Males 90 g  Females 75 g | 8 weeks | Fermented noodles (*L. plantarum*) | Wheat noodles | Muscle mass |
| Huang 2019 a | 9 x 10^10^ CFU | 6 weeks | *L. Plantarum* TW10K | Placebo | Muscle mass |
| Huang 2019 b | 6 x 10^10^ CFU | 4 weeks | *L. plantarum* PS128 and concurrent training | Placebo and training | Muscle mass |
| Skrypnik 2019 | 10^10^CFU | 12 weeks | *Bifidobacterium bifidum* W23, *B.lactis* W51. *B. lactis* W52. *Lactobacillus acidophilus* W37. *Lactobacillus brevis* W63. *Lactobacillus casei* W56, *Lactobacillus salivarius* W24. *Lactococcus lactis* W19 and *Llactis* W58 | Placebo | Lean Body Mass |
| Inoue 2018 | 1.25 x 10^10^ CFU each strain | 12 weeks | 1. *longum* BB536.   *Bifidobacterium infantis* M-63, *B.breve M-16V* and *B. breve B-3* and RT | Placebo | Lean Body Mass |
| Nilsson 2018 | 10^10^ CFU | 48 weeks | *Lactobacillus reuteri* | Placebo | Lean Body Mass |
| Minami 2018 | 2 x10^9^ CFU | 12 weeks | *B. breve* B-3 | Placebo | Muscle mass |
| Szulinska 2018 | 10^10^ CFU | 12 weeks | *B.bifidum* W23. *B.lactis* w51. *B.lactis* w52, *L.acidophilus* W37. *L.brevis* W63. *L.casei* W56. *L.salivarius* W24, *L.lactis* W19 and *L.lactis* w58 | Placebo | Lean Body Mass |
| Toohey 2020 | 5 x 10^9^CFU | 10 weeks | *Bacillus subtilis* (DE111) and recovery drink (45 g CHO, 20 g protein, 2 g fat) | Placebo and recovery drink | Lean Body Mass  Global muscle strength |
| Kim 2018 | 10^10^ CFU | 12 weeks | *Lactobacillus gasseri* BNR17 and mild energy restriction and increased physical activity | Placebo and mild energy restriction and physical activity | Lean Body Mass |
| Osterberg 2015 | 9 x 10^10^ CFU | 4 weeks | *S.thermophilus* DSM24731.  *L.acidophilus* DSM24735.  *L.delbrueckii ssp.* Bulgaricus DSM24734,  *L.paracasei* DSM24733,  *L.plantarum* DSM24730.  *B.lonqum* DSM24736.8. infantis DSM24737 and *B.breve* DSM24732 and high-fat and hypocaloric diet | Placebo | Lean Body Mass |
| Minami 2015 | 5 x 10^10^ CFU | 12 weeks | *B.breve* B-3 | Placebo | Muscle mass |
| Sharafedtinov 2013 | 50 g to 1.5 x 10^11^ CFU | 3 weeks | Cheese (*L.plantarum* TENSlA) and hypocaloric diet | Placebo and hypocaloric diet | Muscle mass |
